# Seasonal Variability and Sex-Specific Accumulation of Trace Metals in Black Scorpionfish (*Scorpaena porcus* Linnaeus, 1758) from Izmir Bay (Aegean Sea), Türkiye: Implications for Human Health Risk Assessment

**DOI:** 10.3390/life15030501

**Published:** 2025-03-20

**Authors:** Mine Percin Olgunoglu, Ilkan Ali Olgunoglu, Engin Artar

**Affiliations:** Veterinary Department Adiyaman, Kahta Vocational Training School, Adiyaman University, 02040 Adiyaman, Türkiye; ilkanali@yahoo.com (I.A.O.); enginartar@hotmail.com (E.A.)

**Keywords:** Aegean Sea, black scorpionfish (*Scorpaena porcus*), heavy metal accumulation, human health risk, Izmir Bay, seasonal variation

## Abstract

This study investigates seasonal and gender-related variations in some metal (Cd, Cr, Cu, Fe, Hg, Mn, Ni, Pb, Se, and Zn) accumulation in black scorpionfish (*Scorpaena porcus*) from Izmir Bay (Aegean Sea, Türkiye) and assesses the associated human health risks. Samples, collected across four seasons from 2023 to 2024, were analyzed for element concentrations using inductively coupled plasma mass spectrometry (ICP-MS). Health risks were calculated using several models, including estimated weekly intake (EWI), target hazard quotient (THQ), total THQ (∑THQ), carcinogenic risk (CR), Se health benefit value (HBVSe), and the Metal Pollution Index (MPI). The results indicate that the consumption of black scorpionfish poses potential health risks, primarily due to the accumulation of manganese (Mn), lead (Pb), and mercury (Hg), which exceeded international permissible legal limits. Gender-based differences were observed, with males showing higher accumulation (*p* > 0.05); however, these differences were not statistically significant. In contrast, significant sex-based differences were identified specifically for cadmium (Cd), with concentrations exhibiting a statistically meaningful difference (*p* < 0.05). Seasonal variations were also apparent (*p* < 0.05). The carcinogenic risk (CR) analyses revealed that chromium (Cr) and nickel (Ni) levels surpassed carcinogenic risk thresholds. Furthermore, the MPI values provided additional insights into the overall metal pollution levels in the fish. These findings underscore the critical importance of monitoring metal pollution, revising fishery management strategies, and managing fish consumption to safeguard public health.

## 1. Introduction

In many fast-growing cities around the world, marine ecosystems face alarming levels of heavy metal contamination, posing significant threats to both human health and marine biodiversity [1,2].

Heavy metals are introduced into marine environments primarily through industrial discharges and agricultural runoff. These pollutants accumulate in aquatic organisms, creating health risks for humans who consume seafood regularly. The persistence of these metals in the environment, coupled with their long biological half-lives, necessitates continuous monitoring of their levels in marine biota [3,4].

Among various marine organisms, fish are particularly susceptible to heavy metal contamination. These pollutants can be absorbed from multiple sources, including water, sediment, and food, resulting in concentrations within fish tissues that far exceed those found in their surrounding environment [5,6,7]. Certain metals, including mercury (Hg), lead (Pb), and cadmium (Cd), are of particular concern due to their high toxicity and their tendency to accumulate in edible fish tissues, posing risks even at trace levels. Research indicates that exposure to these metals can lead to serious health issues, including cognitive dysfunction, hematological disorders, and damage to vital organs like the liver, kidneys, and lungs [8]. This is especially concerning in rapidly developing coastal areas like the Türkish Aegean Sea, which faces increasing contaminant discharges due to demographic expansion and ongoing urban and industrial development [9]. Given these health risks and environmental pressures, it is crucial to investigate the specific impacts of heavy metal accumulation in marine species within this region. The Izmir Bay is an integral part of the Aegean Sea ecosystem, possessing significant biological diversity and economic value. However, the environmental status of this region and heavy metal contamination are issues that require attention and monitoring. This study aims to evaluate the accumulation of heavy metals in the muscle tissues of male and female *Scorpaena porcus* collected from Izmir Bay, specifically focusing on the concentrations of ten metals—Cd, Cr, Cu, Fe, Hg, Mn, Ni, Pb, Se, and Zn—across different seasons.

Black scorpionfish (*S. porcus*), a demersal, carnivorous, traumatogenic, and non-migratory (habitat-restricted) fish species, is widely distributed in the Eastern Atlantic from the British Isles to Morocco and throughout the Mediterranean Sea and Black Sea. Within Türkiye, its primary habitat is the Aegean Sea [10,11,12]. The total catch was reported to be 134 tons in 2023, highlighting its economic relevance [13]. Its relatively slow growth rate [12] may contribute to its potential for accumulating significant levels of toxic elements. Given the premium market value of scorpion fish, which are commercially valuable for artisanal fishers in the Aegean Sea’s fishing industry [12,14], as well as their role as bioindicators of trace element contamination in coastal fish species [15], understanding the extent of heavy metal contamination in their tissues is crucial for both ecological and economic considerations.

Seasonal variations in heavy metal concentrations are particularly critical, as environmental and biological factors influencing metal cycles in aquatic ecosystems can significantly affect the levels of metal accumulation in organisms. By analyzing these seasonal variations, this research seeks to assess human health risks associated with metal contamination while contributing to the broader field of marine pollution studies. The findings are expected to provide essential data for public health policies aimed at promoting ecosystem integrity and safeguarding human well-being.

## 2. Materials and Methods

### 2.1. Sample Collection and Preparation

Izmir Bay, situated on the western coast of the Aegean Sea between latitudes 38°20′ and 38°42′ N and longitudes 26°25′ and 27°10′ E, lies within Türkiye [16], a country surrounded by four different seas, where developing fisheries contribute significantly to the national and local economies. The Aegean Sea, a vital part of the Mediterranean, stretches along Türkiye’s southwestern coast from the Dalaman River to the Greek border, encompassing 2805 km of coastline characterized by a mosaic of bays, estuaries, deltas, and crucial fishing grounds. Recognizing the Aegean’s significant contribution to Türkiye’s fisheries [17,18], and the importance of maintaining its health and productivity, this research therefore focuses on the seasonal and sex-specific accumulation of trace metals in black scorpionfish in Izmir Bay, with an assessment of potential human health risks. Black scorpionfish were purchased seasonally from Limited Liability Izmir Güzelbahçe Fisheries Cooperative during 2023 and 2024. A total of 52 specimens were collected across all seasons. The collected specimens showed a gender distribution of 54% males and 46% females. The seasonal sampling periods were as follows: first group, autumn (25–30 November 2023); second group, winter (5–10 February 2024); third group, spring (27 March–3 April 2024); fourth group, summer (10–14 September). Sampling was not conducted during the hunting ban periods for marine fish in Türkiye, which typically occurs from 15 April to 1 September.

To preserve sample integrity during transport to the laboratory, samples were placed in insulated containers packed with crushed ice. Upon arrival at the laboratory, total length and weight measurements were recorded for each specimen. Following this, during the gender determination process, the head, fins, scales, skin, gonads, and all internal organs, along with muscle tissue, were removed. Edible muscle tissue was then dissected, placed in labeled polyethylene bags, and stored at −20 °C until further processing for metal analysis.

### 2.2. Determination of Elements

To maintain sample integrity, the samples were transported on dry ice to the laboratory where the analysis was conducted.

### 2.3. Microwave Digestion Procedure

Microwave digestion was performed using a Berghof MSW-4 microwave digestion system. Approximately 250 mg (wet weight) of fish tissue samples were weighed and transferred into digestion vessels. To each sample, 5 mL of 65% HNO_3_ (nitric acid) and 1 mL of 37% HCl (hydrochloric acid) were added. The vessels were gently shaken and left uncovered for 15–20 min to allow initial reactions to subside. The digestion process was conducted according to the following program:

The temperature was increased to 160 °C over 5 min.
The samples were held at 160 °C for 5 min.
The temperature was then increased to 190 °C over 1 min.
The samples were held at 190 °C for 15 min.
Finally, the samples were cooled to 50 °C.

The digestion parameters were set to a maximum pressure of 40 bar and 80% power during the heating steps. After digestion, the samples were allowed to cool to room temperature.

### 2.4. ICP-MS Analysis

Elemental analysis was carried out using a Perkin Elmer NexION 350X ICP-MS instrument. The system was operated under the following conditions:

Nebulizer: Mainhard (concentric).
Spray chamber: glass cyclonic.
Triple-cone interface material: nickel.
Plasma gas flow rate: 18.0 L/min.
Auxiliary gas flow rate: 1.2 L/min.
Nebulizer gas flow rate: 0.76 L/min.
Sample uptake rate: 1 mL/min.
RF power: 1500 W.
Replicates per sample: 3.
Mode of operation: standard (STD) mode and kinetic energy discrimination (KED) mode.
Collision gas: helium (He).
Sample preparation for ICP-MS.

After digestion, the final volume of each sample was adjusted to 10 mL with ultra-pure water. From this solution, 0.5 mL was extracted and further diluted as required to ensure compatibility with the ICP-MS system. All samples were analyzed in triplicates to ensure the reproducibility and accuracy of the results, which are presented in mg kg^−1^. The accuracy and precision of our results were validated through the analysis of the standard reference material, NIST 2976 (mussel tissue).

### 2.5. Evaluation of Health Risk

The health risk associated with heavy metal consumption was evaluated using the following equations:

Equation (1): estimated daily intake (EDI) of heavy metals [19]:

(1) $$EDI=\frac{MC\times FDC}{BW}$$

MC (mg/kg) is the concentration of heavy metals in fish muscle.

FDC is the average daily demersal fish consumption (3.31 g/person/day in Türkiye) [20].

BW is the average body weight (70 kg for adults).

Equation (2): estimated weekly intake (EWI) of heavy metals [21]:

(2) EWI=EDI×7

Equation (3): target hazard quotients (THQs) [19]:

(3) $$THQ=\frac{EF\times ED\times FIR\times C\times C}{RfDs\times BW\times ATn}\times 10^{-3}$$

Equation (4): total target hazard quotient (∑THQ) [22]:

∑THQ = THQ(Zn) + THQ(Mn) + … THQ(Hg)(4)

A THQ or ∑THQ value of ≤1 is defined as an acceptable risk level, indicating no significant non-carcinogenic health risks. Conversely, values exceeding 1 imply potential health risks for consumers [23,24].

Factors, units, and values in the target hazard quotient (THQ) formula are provided in Table 1.

Equation (5): target carcinogenic risk (CR).

Among the analyzed heavy metals, such as Cd, Cr, Ni, and Pb—all classified as carcinogens by the International Agency for Research on Cancer (IARC)—this risk is calculated based on their average concentration in samples. The target carcinogenic risk (CR) equation, as referenced by Islam et al. [30], Tokatlı and Ustaoglu [31], and Alam et al. [32], is used to quantify the lifetime cancer risk associated with consuming these contaminants.

(5) $$CR=\frac{EF\times ED\times FIR\times C\times CSF}{BW\times ATn}\times 10^{-3}$$

CSF represents the cancer slope factor. The CSF values for Pb, Cr, Ni, and Cd are 0.0085, 0.5, 0.91, and 0.38 (mg/kg/day), respectively [32,33,34].

### 2.6. The Se Health Benefit Value (HBVSe)

The Se/Hg molar ratio was determined for each tissue sample by dividing the molar concentration of Se by the molar concentration of mercury. Molar concentrations were calculated by dividing the measured concentration (in mg/kg) of each element by its respective atomic weight (Se: 78.96 g/mol; Hg: 200.59 g/mol). The selenium health benefit value (HBVSe) was calculated specifically for edible muscle tissue using the equation described by Osuna et al. [35]:

HBVSe = ((Se) − (Hg))/(Se) × ((Se) + (Hg))

### 2.7. Ecological Risk Assessment

The Metal Pollution Index (MPI) evaluates metal contamination in fish muscle by calculating the geometric mean of the concentrations of detected heavy metals. The MPI is calculated using the following formula [8]:

MPI (mg/kg) = (M_Zn_ × M_Mn_ × M_Cu_ × … … M_Hg_)^(1/n)^

### 2.8. Statistical Analysis

Statistical analyses were performed using SPSS version 21.0 for Windows (IBM Corp., Armonk, NY, USA). Data are presented as mean ± standard deviation (SD). Differences among group means were assessed using one-way analysis of variance (ANOVA). When ANOVA revealed significant differences (*p* < 0.05), post hoc comparisons were conducted using Duncan’s multiple range test.

## 3. Results and Discussion

### 3.1. Average Weights and Total Lengths of Fish Samples

Table 2 presents average weights (g) and total lengths of male and female fish species collected from Izmir Bay (Aegean Sea), Türkiye.

The statistical analysis indicated that the differences in total length and weight between female and male black scorpionfish were not statistically significant (*p* > 0.05). Additionally, no significant differences in the weight or length were detected among the seasons (*p* > 0.05).

Akalın et al. [36] reported that the length values of *S. porcus* individuals in their monthly study in Izmir Bay varied between 7.5 and 27.2 cm. The values obtained in our study fall within the range reported by the researchers, confirming the consistency of our findings.

### 3.2. Metal Concentrations of Samples

Biological differences within species, such as growth rate, feeding strategies (including feeding rate, assimilation efficiency, and composition), and the contribution of organic material, appear to be important factors determining elemental accumulation [37]. Table 3 summarizes the sex-based seasonal variations in metal concentrations (mg kg^−1^ wet weight) within the edible muscle of black scorpionfish. The statistical analysis revealed significant seasonal differences in the concentrations of Cu, Cr, Fe, Hg, Mn, Pb, Se, and Zn (*p* < 0.05). In contrast, Cd and Ni concentrations did not show significant seasonal variation (*p* > 0.05). Sex-based differences were observed only for Cd, with concentrations differing significantly (*p* < 0.05) between male and female fish all seasons. No other metals exhibited significant sex-based variations (*p* > 0.05). These sex-based differences are consistent with those of Bat et al. [38], who investigated heavy metal concentrations in the edible tissues of male and female turbot (*Scophthalmus maximus*) from the Black Sea coast, examining mean concentrations of Cu, Cd, Fe, Hg, Pb, and Zn Similar to the present study, they reported no significant differences between male and female fish for most metals, except for Cd.

The following sections detail the seasonal and gender-based variations in metal bioaccumulation, as presented in Table 3.

Cd (cadmium): Cd, a non-essential and potentially toxic element, is known to bioaccumulate in organisms [41]. In this study, the Cd concentrations in black scorpionfish remained relatively consistent across seasons and sexes, although minor fluctuations were observed. In females, the highest Cd concentration (0.042 mg kg^−1^) was measured in autumn, while the lowest (0.027 mg kg^−1^) was recorded in summer. The male Cd levels ranged from 0.044 mg kg^−1^ in spring to 0.038 mg kg^−1^ in autumn. All measured Cd concentrations were well below the maximum permissible level of 0.500 mg kg^−1^ wet weight established by the Food and Agriculture Organization (FAO) for fish [39].

Cr (chromium): Cr is a biologically active element that plays a role in various metabolic processes [42]. In females, the highest Cr concentration was recorded in spring (0.457 mg kg^−1^), while the lowest was in summer (0.097 mg kg^−1^). For males, Cr levels ranged from 0.474 mg kg^−1^ in autumn to 0.144 mg kg^−1^ in spring.

Cu (copper): Cu is an essential trace element that plays a vital role in various biological processes but can be toxic in excessive amounts [43]. The Cu levels in females were highest in spring (0.977 mg kg^−1^) and lowest in summer (0.237 mg kg^−1^). In males, Cu concentrations ranged from 0.605 mg kg^−1^ in summer to 0.292 mg kg^−1^ in winter. The permissible limit for Cu is established at 20 mg kg^−1^ [44]. Notably, the Cu concentrations observed in both females and males are consistently below this limit.

Fe (iron): Fe is an abundant element on Earth and is a biologically essential component for all living organisms [45]. The Fe concentrations were higher in females during spring (58.670 mg kg^−1^) and lowest in summer (20.829 mg kg^−1^). For males, the Fe levels peaked in winter (58.685 mg kg^−1^) and were lowest in autumn (34.452 mg kg^−1^). The permissible limit for Fe is established at 100 mg kg^−1^ [40]. All observed Fe concentrations in both females and males are below this threshold across all seasons.

Hg (mercury): Hg is a highly toxic metal pollutant that is often introduced into aquatic environments through industrial and agricultural discharges [41]. In this study, the Hg concentrations in the muscle of black scorpionfish exhibited statistically significant seasonal variations (*p* < 0.05). Females accumulated the highest Hg concentration in summer, measuring 1.482 mg kg^−1^, which exceeds the legal EU maximum limit of 1.000 mg kg^−1^ for fish muscle [39]. In winter, the lowest levels were observed at 0.074 mg kg^−1^. In males, the Hg concentrations ranged from 0.435 mg kg^−1^ in summer to 0.106 mg kg^−1^ in spring, remaining below the EU limit. These findings indicate a potential risk for consumers. High Hg values may occur due to natural and anthropogenic riverine inputs. The effluent from large chlor-alkali and chlorine plants likely supplies most of the Hg found in the surface sediments of inner Izmir Bay [46]. The demersal lifestyle of black scorpionfish increases their direct exposure to mercury accumulated in the sediments.

Mn (manganese): Mn is a relatively abundant metal, widely distributed in the Earth’s crust and found in nodules on the seafloor [47]. In females, the highest Mn concentration was observed in spring at 1.976 mg kg^−1^, while the lowest was recorded in summer at 0.196 mg kg^−1^. For males, Mn concentrations were highest in autumn at 1.056 mg kg^−1^ and lowest in spring at 0.577 mg kg^−1^. The permissible limit for Mn is set at 1.00 mg kg^−1^ [40]. It is notable that both female black scorpionfish in spring (1.976 mg kg^−1^) and male black scorpionfish in autumn (1.056 mg kg^−1^) exhibit Mn concentrations exceeding the permissible limit. The potential reasons for these elevated Mn levels include the reduction of oxygen during the decomposition of organic matter in the inner bay sediments, which plays a significant role in the dissolution of manganese oxides [48]. In this context, the demersal lifestyle of the black scorpionfish directly exposes it to the Mn accumulation in the sediments, thus contributing to the high Mn concentrations observed.

Ni (nickel): Ni can be essential or toxic in ecosystems, based on its concentration [49]. With the exception of spring, the Ni levels were generally higher in males compared to the females. In females, the highest Ni concentration was observed in spring (1.464 mg kg^−1^), while the lowest occurred in winter (0.172 mg kg^−1^). For males, the Ni levels peaked in winter (2.494 mg kg^−1^) and were lowest in spring (0.190 mg kg^−1^).

Pb (lead): Pb, a harmful metal with no known biological function, enters aquatic environments through human activities [41]. Its lipophilic nature allows fish to readily absorb and accumulate it in their blood and bones [1]. In females, the highest Pb concentration was observed in spring (0.429 mg kg^−1^), while the lowest occurred in autumn (0.323 mg kg^−1^). For males, Pb levels ranged from 0.404 mg kg^−1^ in spring to 0.376 mg kg^−1^ in summer. All measured Pb concentrations in both female and male fish exceeded the maximum permissible level of 0.300 mg kg^−1^ established by the Food and Agriculture Organization (FAO) [39], indicating potential environmental contamination. This contamination is consistent with findings that Pb is very heavily enriched in the inner Izmir Bay (Özkan, 2012). While high enrichment factors often suggest anthropogenic contributions, natural sources can also contribute to the observed enrichments [46]. Therefore, the elevated Pb levels in the fish may result from a combination of human-induced pollution and natural geological sources within the bay.

Se (selenium): Se is an essential trace element vital for human homeostasis and immune function, known for its antioxidant properties and as a cofactor for enzymes like glutathione peroxidase [50]. In females, the highest Se concentration was recorded in summer (0.631 mg kg^−1^), while the lowest was in winter (0.302 mg kg^−1^). For males, the Se levels were highest in summer (0.952 mg kg^−1^) and lowest in winter (0.416 mg kg^−1^).

Zn (zinc): Zn is an essential mineral for organisms, a biological trace element required daily for optimal health. Despite its vital importance, Zn can become toxic when its levels exceed the maximum allowable value [44,51]. In females, the highest Zn concentration was observed in autumn (4.206 mg kg^−1^), while the lowest was recorded in winter (2.582 mg kg^−1^). For males, Zn concentrations peaked in spring (5.741 mg kg^−1^) and were lowest in winter (2.806 mg kg^−1^). The permissible limit for Zn is set at 40 mg kg^−1^ [40]. All observed Zn concentrations in both females and males across all seasons are well below this limit.

Overall, in this study, male black scorpionfish exhibited higher levels of element accumulation compared to the females, a trend that was particularly pronounced during the winter season. These findings are consistent with previous research, such as that reported by Dirican [52], which also observed higher accumulation in male individuals. These differences in accumulation due to gender may be metal-specific, indicating that the accumulation patterns can vary not only between males and females but also depending on the type of elements present [52]. Additionally, the observed differences in the levels of heavy metals in black scorpionfish likely reflect a complex interplay of factors, including environmental conditions (e.g., water temperature, salinity, pH, and food availability), dietary habits (e.g., changes in prey availability and composition), and the physiological state of the fish, such as reproductive cycles (e.g., vitellogenesis in females) and growth patterns [38]. In conclusion, this complexity underscores the need to consider both seasonal and gender-based factors when assessing heavy metal accumulation in marine organisms.

The decreasing order of seasonal and gender-based mean metal concentrations in the edible muscle of black scorpionfish was as follows:

In the autumn for females: Fe (29.189 mg kg^−1^) > Zn (4.206 mg kg^−1^) > Mn (0.890 mg kg^−1^) > Cu (0.539 mg kg^−1^) > Se (0.508 mg kg^−1^) > Pb (0.323 mg kg^−1^) > Cr (0.213 mg kg^−1^) > Ni (0.212 mg kg^−1^) > Hg (0.131 mg kg^−1^) > Cd (0.042 mg kg^−1^).

Males: Fe (34.452 mg kg^−1^) > Zn (3.520 mg kg^−1^) > Mn (1.056 mg kg^−1^) > Se (0.622 mg kg^−1^) > Ni (0.645 mg kg^−1^) > Cu (0.492 mg kg^−1^) > Cr (0.474 mg kg^−1^) > Pb (0.380 mg kg^−1^) > Hg (0.324 mg kg^−1^) > Cd (0.038 mg kg^−1^).

In the winter for females: Fe (57.281 mg kg^−1^) > Zn (2.582 mg kg^−1^) > Mn (0.559 mg kg^−1^) > Pb (0.371 mg kg^−1^) > Se (0.302 mg kg^−1^) > Cu (0.245 mg kg^−1^) > Cr (0.183 mg kg^−1^) > Ni (0.172 mg kg^−1^) > Hg (0.074 mg kg^−1^) > Cd (0.033 mg kg^−1^).

For males: Fe (58.685 mg kg^−1^) > Zn (2.806 mg kg^−1^) > Ni (2.494 mg kg^−1^) > Mn (0.676 mg kg^−1^) > Se (0.416 mg kg^−1^) > Pb (0.398 mg kg^−1^) > Hg (0.379 mg kg^−1^) > Cu (0.292 mg kg^−1^) > Cr (0.239 mg kg^−1^) > Cd (0.040 mg kg^−1^).

In the spring for females: Fe (58.670 mg kg^−1^) > Zn (3.453 mg kg^−1^) > Mn (1.976 mg kg^−1^) > Ni (1.464 mg kg^−1^) > Cu (0.977 mg kg^−1^) > Pb (0.429 mg kg^−1^) > Cr (0.457 mg kg^−1^) > Se (0.456 mg kg^−1^) > Hg (0.157 mg kg^−1^) > Cd (0.034 mg kg^−1^).

For males: Fe (49.440 mg kg^−1^) > Zn (5.741 mg kg^−1^) > Mn (0.577 mg kg^−1^) > Se (0.497 mg kg^−1^) > Pb (0.404 mg kg^−1^) > Cu (0.295 mg kg^−1^) > Ni (0.190 mg kg^−1^) > Cr (0.144 mg kg^−1^) > Hg (0.106 mg kg^−1^) > Cd (0.044 mg kg^−1^).

In the summer for females: Fe (20.829 mg kg^−1^) > Zn (3.526 mg kg^−1^) > Hg (1.482 mg kg^−1^) > Ni (0.691 mg kg^−1^) > Se (0.631 mg kg^−1^) > Pb (0.349 mg kg^−1^) > Cu (0.237 mg kg^−1^) > Mn (0.196 mg kg^−1^) > Cr (0.097 mg kg^−1^) > Cd (0.027 mg kg^−1^).

For males: Fe (47.798 mg kg^−1^) > Zn (2.955 mg kg^−1^) > Mn (1.001 mg kg^−1^) > Se (0.952 mg kg^−1^) > Ni (0.923 mg kg^−1^) > Cu (0.605 mg kg^−1^) > Hg (0.435 mg kg^−1^) > Pb (0.376 mg kg^−1^) > Cr (0.213 mg kg^−1^) > Cd (0.039 mg kg^−1^).

In the present study, Fe and Zn were identified as the most prevalent metals in the muscle tissue of black scorpionfish (*S. porcus*). While Cd was consistently detected, it was present at significantly lower concentrations than the other metals. This dominance of Fe and Zn in the muscle tissue aligns with previous findings [38,53,54]. Furthermore, Ahmed et al. [55] reported that Fe is the most abundant metal in various fish species across different seasons, likely reflecting its essential biological roles. As a crucial component of hemoglobin, Fe is often found in excess in tissues [55], and its higher accumulation in living organisms is considered a normal physiological process [51]. In addition, comparing our results with those of Çulha et al. [3] for the same species in the Black Sea reveals notable differences in the concentrations of several metals. Çulha et al. [3] reported the following concentrations (mg kg^−1^): Cu as 0.07 ± 0.009; Ni as 0.01 ± 0.07; Cd as not detected, Hg as 0.01 ± 0.00, and Pb as 0.04 ± 0.03. Additionally, Bat et al. [56] reported concentrations for the same species and region: Hg at 0.012, Cd at 0.01, Pb at 0.02, Cu ranging from 0.05 to 0.14, and Zn ranging from 0.9 to 3.22 mg/kg. In the present study, the determined concentrations of these metals were generally higher. In another study by Ourgaud et al. [15], which examined trace element concentrations, bioaccumulation processes, and spatial variations in black scorpionfish from three sites within the Bay of Marseille in the Mediterranean Sea, the following trace element concentrations were reported (mg/kg): Cd as 0.029 ± 0.010, 0.025 ± 0.012, and 0.021 ± 0.014; Cr as 0.64 ± 0.28, 2.72 ± 4.16, and 1.75 ± 0.94, Cu as 0.58 ± 0.01, 0.50 ± 0.15, and 0.60 ± 0.10; Hg as 0.70 ± 0.47, 1.03 ± 1.01, and 0.93 ± 0.86; Ni as 0.33 ± 0.05, 0.36 ± 0.39, and 0.52 ± 0.32; Pb as 0.029 ± 0.010, 0.025 ± 0.012, and 0.028 ± 0.019; Zn as 48.66 ± 5.45, 61.62 ± 12.95, and 46.89 ± 15.85. Notably, the Zn values reported in the present study are significantly lower than those of Ourgaud et al. [15]. All these discrepancies are likely attributable to geographical variations in environmental conditions between the respective sampling locations.

### 3.3. Comparison of Metal Levels with Previous Studies from the Aegean Sea

Table 4 presents the levels of metal concentrations and the reflection of contamination in various fish species from the Aegean Sea based on data from national and international studies conducted over the past ten years.

The comparison of average metal concentrations in fish species shows variations in metal levels among different species (Table 4).

For instance, the Cd values observed in this study, with the exception of the values reported by Topal and Onac [61], as 5.40 ± 0.25 for *Mugil cephalus* and 4.96 ± 0.24 for *Sparus aurata,* were generally close to the average reported values. The maximum Cr values detected in this study were higher than the average Cr levels reported for other species, whereas the minimum Cr values were lower. The Cu concentrations identified in this study were within the range of average values reported for other species.

In contrast, the Fe levels, except for the average values reported by Ateş et al. [57], were generally found to be higher. The Mn concentrations were observed to be lower than those reported in previous studies. The Ni concentrations, except for the value of 4.036 ± 0.68 reported by Topal and Onac [61] for *S. aurata*, were generally higher.

The Pb concentrations measured in this study were slightly elevated compared to the values reported in the literature, while the Zn concentrations were slightly lower. Finally, the Hg concentrations were generally found to be higher compared to the values reported by other researchers.

In conclusion, previous studies have shown that a range of factors can influence the accumulation of metals in fish tissues. These factors include variations among species, seasonal fluctuations, the size and weight of the fish, differing ecological needs, metabolic activities, feeding behaviors, as well as the physical and chemical characteristics of the water and levels of pollution. For example, bottom-dwelling (benthic) fish tend to accumulate higher levels of metals compared to open-water (pelagic) species. This increased accumulation in benthic species is largely due to the higher concentration of metals found in sediment, where they live and feed [1].

### 3.4. Health Risk Assessment

Sometimes, even though fish muscle generally has low metal levels, the potential risk can differ based on how much is consumed [24].

Table 5 compares the estimated weekly intakes (EWIs) of selected metals to their corresponding provisional tolerable weekly intakes (PTWIs), which represent safe lifetime exposure levels set by the Joint Expert Committee on Food Additives (JECFA) under the FAO and WHO. PTWI values are based on factors such as food consumption, duration, and contamination levels [21].

While the EWIs for Cu, Mn, and Zn were well below PTWI limits, indicating safe intake levels, exceedances were observed for Cd, Cr, Fe, Hg, Pb, Ni, and Se. Among these, Fe showed the highest EWI values, but all exceeded their respective PTWIs, highlighting potential health risks associated with consuming black scorpionfish. These consistent exceedances across multiple metals underscore the need for caution regarding consumption from the Aegean Sea. EWI of a substance from a food source is not enough to determine if that food is safe to eat [65]. Thus, the THQ values were computed (Table 6).

Table 6 presents the target hazard quotient (THQ) and total THQ (∑THQ) for elements in the muscle tissue of male and female scorpionfish, which are crucial indicators for assessing potential non-carcinogenic health risks associated with consumption. The THQ values quantify the risk posed by individual heavy metals, while ∑THQ provides an integrated assessment of cumulative risk from multiple contaminants. These metrics are essential for evaluating the safety of scorpionfish consumption, particularly in regions where they are a significant dietary component.

In this study, THQ values for all elements and seasons remained below 1 for adults (Table 6), indicating no immediate non-carcinogenic risk. However, during the summer months, THQ values for the female scorpionfish approached 1, reaching 0.7, suggesting a potential increase in risk during this period. The summer ∑THQ value of 0.7182, primarily attributable to the Hg levels, reinforces this observation. This finding aligns with previous research by Stamatis et al. [39], which reported Hg THQ values exceeding 1 in albacore tuna (*Thunnus alalunga*) from the North Aegean Sampling Station Area (NASSA) and Southeastern Aegean Sampling Station Area (SASSA) in Greece, with ranges of 1.338–5.040 and 0.758–4.046, respectively. Furthermore, while Stamatis et al. [39] reported Cd THQ values ranging from 0.012 to 0.359 in NASSA and 0.011 to 0.316 in SASSA, they did not report Pb THQ values exceeding 1. However, their reported total THQs (TTHQs) for albacore tuna exceeded unity, ranging from 1.353 to 5.213 in NASSA and 0.078 to 4.058 in SASSA. These elevated TTHQ values underscore the potential for adverse health effects in consumers of Aegean Sea seafood, highlighting the importance of considering cumulative metal exposure. The CR values exceeding 10^−^^4^ suggest a potential risk of carcinogenesis, while values between 10^−^^4^ and 10^−^^6^ indicate that the carcinogenic risk is considered acceptable. In contrast, CR values below 10^−^^6^ signify a very low risk of carcinogenesis [33].

Table 7 presents the results of the target carcinogenic risk (CR) assessment for Cd, Cr, Ni, and Pb.

As shown in Table 7, Cd values ranged between 10^−4^ and 10^−6^, remaining within acceptable risk limits. This suggests that the carcinogenic effect of the Cd in these samples is relatively low. However, Cr concentrations exceeded the 10^−4^ threshold, indicating a potential risk. These results suggest that the Cr exposure may be a risk factor for cancer development. The Pb values were found to be close to the “potential risk” threshold, indicating that lead concentrations also require careful monitoring. These findings underscore the importance of monitoring heavy metal risks across different environments. In this context, a study on health risks associated with heavy metal ingestion from two predominant fish species in a developing country, the carcinogenic risk (CR) values for Cr were within the acceptable range, indicating negligible carcinogenic risk for adults. However, Cd exhibited a CR value exceeding the acceptable threshold, suggesting a potential carcinogenic hazard. This indicates that long-term intake of these studied fish increases the probability of suffering from cancer-related diseases [66]. These varying risk profiles in fish necessitate the examination of heavy metal risks in other environmental samples.

Similarly, the Ni values were significantly above the 10⁻⁴ limit, placing them in the “potential risk” category. This finding suggests that the carcinogenic effect of Ni in these samples may be high. In light of these findings, this study demonstrates that the concentrations of the examined toxic elements can pose varying levels of carcinogenic risk. High concentrations of Cr and Ni, in particular, highlight the importance of continuous monitoring of environmental heavy metal levels and the implementation of proactive measures.

### 3.5. The HBVSe and MPI

A positive HBVSe value indicates that the molar concentration of Se is greater than that of Hg, suggesting a potential health benefit to consumers. Conversely, a negative HBVSe value indicates a higher molar concentration of mercury than Se (Table 8).

In our study, Hg levels frequently exceeded the 1.00 mg/kg limit established by international authorities. Consequently, although the selenium health benefit value (HBVSe) was calculated, its interpretation should be considered in light of the elevated Hg concentrations. It is thought that Hg toxicity poses a primary health risk, and therefore, evaluating the potential benefits of the Se in this context may not be entirely accurate.

Metals can be absorbed by fish through their diet or direct contact with polluted water on their respiratory surfaces [67]. To investigate bioaccumulation trends of ten metals and assess pollution levels in the studied organisms (Table 8), the Metal Pollution Index (MPI) was calculated. An MPI value greater than 1 indicates contamination, while values below 1 suggests no contamination [60]. In this study, all MPI values were less than 1, indicating no contamination based on this metric. While males generally exhibited higher MPI values than females, this trend reversed in spring, with females showing elevated MPI values. The highest MPI, recorded in spring for females, and the lowest in winter, suggest a potential seasonal influence on metal bioaccumulation. Similar seasonal trends were reported by Omar et al. [68], where the highest MPI values were observed in spring and the lowest in winter. Furthermore, a study by Kalipci et al. [69] on the toxicological health risk analysis of hazardous trace element accumulation in five commonly consumed fish species in Türkiye also reported MPI values less than 1, indicating a low risk of contamination in those species as well.

## 4. Conclusions

This study clearly demonstrates that the consumption of black scorpionfish from Izmir Bay carries potential human health risks due to the accumulation of certain heavy metals, particularly Cr, Hg, Mn, Ni, and Pb. These findings highlight the impact of localized pollution in Izmir Bay and reveal that metal concentrations in the fish exceed established safety limits. The observed gender-based differences, with higher accumulation of some toxic elements in males, and seasonal variations, such as elevated the Hg levels in females during the summer, warrant further investigation into the underlying physiological and environmental mechanisms. Although the overall Metal Pollution Index (MPI) did not indicate widespread contamination, the exceedance of provisional tolerable weekly intake (PTWI) values for multiple metals, and the potential carcinogenic risks associated with Cr and Ni in particular, underscore the need for cautious consumption of this fish species. The exceedance of permissible limits for Cr, Hg, Ni, and Pb is of particular concern.

## Figures and Tables

**Table 1:** Parameters and values used in THQ analysis [19,22,25,26,27,28,29].

Statement (Factors; Unit)	Value for Adult
Exposure frequency (EF; days per year)	365
Exposure duration (ED; Years)	70
Food ingestion rate (FIR; g/person/day)	3.31
Metal concentration (C; mg kg^−1^)	Present study
Body weight (BW; Kg)	70
Average exposure time for non-carcinogenic effects (ATn; days per year × ED)	365 × 70
Oral reference dose (RfDs; mg kg^−1^/day)	Cd = 0.001; Cr = 0.003; Cu = 0.04; Fe = 0.7; Hg = 0.0001; Mn = 0.14; Ni = 0.02; Pb = 0.0035; Se = 0.005; Zn = 0.3

**Table 2:** The average weights (g) and total lengths of female and male fish species (mean ± SD).

Gender	Seasons	Weight (g)	Total Length (cm)
♀	Autumn (8)	228.00 ± 62.33	22.50 ± 1.87
♂	Autumn (5)	258.67 ± 106.23	22.83 ± 3.33
♀	Winter (9)	363.33 ± 102.26	26.45 ± 3.71
♂	Winter (4)	309.83 ± 55.16	25.75 ± 2.38
♀	Spring (6)	222.33 ± 74.84	23.33 ± 2.31
♂	Spring (7)	340.00 ± 80.00	26.33 ± 2.08
♀	Summer (5)	271.22 ± 62.33	24.36 ± 2.50
♂	Summer (8)	302.83 ± 96.23	24.97 ± 2.82

The numbers in parentheses next to the seasons indicate the count of male and female individuals. ♀: female; ♂: male.

**Table 3:** Concentrations of heavy metals in the muscle tissue of female and male black scorpionfish by season (mean ± SD, mg kg^–1^ wet weight).

						Elements					
Gender	Seasons	Cd	Cr	Cu	Fe	Hg	Mn	Ni	Pb	Se	Zn
♀	Autumn	0.042 ± 0.001 ^a^	0.213 ± 0.001 ^b^	0.539 ± 0.005 ^ab^	29.189 ± 0.0109 ^a^	0.131 ± 0.001 ^a^	0.890 ± 0.023 ^ab^	0.212 ± 0.001 ^a^	0.323 ± 0.001 ^a^	0.508 ± 0.004 ^b^	4.206 ± 0.003 ^bc^
♂		0.038 ± 0.004 ^a^	0.474 ± 0.002 ^b^	0.492 ± 0.004 ^ab^	34.452 ± 0.087 ^a^	0.324 ± 0.005 ^a^	1.056 ± 0.003 ^ab^	0.645 ± 0.002 ^a^	0.380 ± 0.017 ^a^	0.622 ± 0.001 ^b^	3.520 ± 0.023 ^bc^
♀	Winter	0.033 ± 0.001 ^a^	0.183 ± 0.001 ^ab^	0.245 ± 0.001 ^a^	57.281 ± 0.006 ^b^	0.074 ± 0.002 ^a^	0.559 ± 0.005 ^a^	0.172 ± 0.001 ^a^	0.371 ± 0.001 ^b^	0.302 ± 0.001 ^a^	2.582 ± 0.001 ^a^
♂		0.040 ± 0.001 ^a^	0.239 ± 0.001 ^ab^	0.292 ± 0.001 ^a^	58.685 ± 0.001 ^b^	0.379 ± 0.006 ^a^	0.676 ± 0.016 ^a^	2.494 ± 0.011 ^a^	0.398 ± 0.002 ^b^	0.416 ± 0.022 ^a^	2.806 ± 0.001 ^a^
♀	Spring	0.034 ± 0.002 ^a^	0.457 ± 0.003 ^ab^	0.977 ± 0.004 ^b^	58.670 ± 0.040 ^b^	0.157 ± 0.004 ^a^	1.976 ± 0.002 ^b^	1.464 ± 0.002 ^a^	0.429 ± 0.005 ^c^	0.456 ± 0.003 ^ab^	3.453 ± 0.001 ^c^
♂		0.044 ± 0.002 ^a^	0.144 ± 0.002 ^ab^	0.295 ± 0.002 ^b^	49.44 ± 0.023 ^b^	0.106 ± 0.003 ^a^	0.577 ± 0.004 ^b^	0.190 ± 0.011 ^a^	0.404 ± 0.002 ^c^	0.497 ± 0.003 ^ab^	5.741 ± 0.001 ^c^
♀	Summer	0.027 ± 0.001 ^a^	0.097 ± 0.001 ^a^	0.237 ± 0.003 ^ab^	20.829 ± 0.016 ^a^	1.482 ± 0.002 ^b^	0.196 ± 0.003 ^a^	0.691 ± 0.002 ^a^	0.349 ± 0.002 ^a^	0.631 ± 0.001 ^c^	3.526 ± 0.003 ^ab^
♂		0.039 ± 0.020 ^a^	0.213 ± 0.001 ^a^	0.605 ± 0.002 ^ab^	47.798 ± 0.004 ^a^	0.435 ± 0.002 ^b^	1.001 ± 0.003 ^a^	0.923 ± 0.004 ^a^	0.376 ± 0.003 ^a^	0.952 ± 0.002 ^c^	2.955 ± 0.002 ^ab^
	PL	0.500	-	20	100	1.00	1.00	-	0.300	-	40
	(IDL)	0.018	0.011	0.011	0.041	0.002	0.038	0.019	0.004	0.101	0.086

♀: female; ♂: male, IDL: instrument detections limit (mg kg^–1^). The data are presented as the mean ± standard deviation of triplicate measurements. Averages that are indicated by different letters in the same row are statistically different from each other (*p* < 0.05); PL: permissible limits (mg kg^−1^) [39,40].

**Table 4:** Concentrations of elements in various fish species from Aegean fishing grounds (mg kg^−1^).

Metals	Species	Concentrations	References	Present Study
Cd	*Serranus cabrilla*	0.20 ± 0.07	[57]	max. 0.044 min. 0.027
	*Diplodus sargus*	0.18 ± 0.06	[57]	
	*Sardina pilchardus*	0.10 ± 0.04	[57]	
	*Spicara maena*	0.06 ± 0.02	[57]	
	*Oblada melanura*	0.03 ± 0.00	[57]	
	*Merluccius merluccius*	0.08 ± 0.03	[57]	
		0.025	[58]	
	*Sardina pilchardus*	0.03 ± 0.01	[59]	
	*Mullus barbatus*	0.03 ± 0.01	[60]	
	*Mullus surmuletus*	0.03 ± 0.01	[60]	
	*Lithognathus mormyrus*	0.03 ± 0.01	[60]	
	*Pagellus erythrinus*	0.03 ± 0.01	[60]	
	*Diplodus vulgaris*	0.03 ± 0.01	[60]	
	*Mugil soiuy*	0.037	[58]	
	*Alosa fallax*	0.015	[58]	
	*Merlangius euxmus*	0.009	[58]	
	*Thunnus alalunga*	0.021–0.669	[37]	
	*Pomatomus saltatrix*	0.011	[58]	
		5.15 ± 0.27	[61]	
	*Dicentrarchus labrax*	0.005	[58]	
		0.24 ± 0.01	[61]	
	*Mugil cephalus*	5.40 ± 0.25	[61]	
	*Sparus aurata*	0.06 ± 0.03	[59]	
		0.027	[58]	
		4.96 ± 0.24	[61]	
		0.01 ± 0.01	[62]	
Cr	*Serranus cabrilla*	0.15 ± 0.06	[57]	max. 0.474 min. 0.097
	*Diplodus sargus*	0.52 ± 0.21	[57]	
	*Sardina pilchardus*	0.38 ± 0.15	[57]	
	*Spicara maena*	0.09 ± 0.01	[57]	
	*Oblada melanura*	0.11 ± 0.04	[57]	
	*Merluccius merluccius*	0.29 ± 0.0	[57]	
	*Mullus barbatus*	0.28 ± 0.07	[60]	
	*Mullus surmuletus*	0.27 ± 0.10	[60]	
	*Lithognathus mormyrus*	0.38 ± 0.11	[60]	
		0.38 ± 0.11	[63]	
	*Pagellus erythrinus*	0.31 ± 0.14	[60]	
		0.39 ± 0.07	[63]	
	*Diplodus vulgaris*	0.39 ± 0.07	[60]	
		0.31 ± 0.14	[63]	
	*E. encrasicolus*	0.03 ± 0.00	[59]	
Cu	*Serranus cabrilla*	1.20 ± 0.27	[57]	max. 0.977 min. 0.237
	*Diplodus sargus*	9.86 ± 3.81	[57]	
	*Sardina pilchardus*	4.79 ± 1.66	[57]	
	*Spicara maena*	1.31 ± 0.52	[57]	
	*Oblada melanura*	0.25 ± 0.04	[57]	
	*Merluccius merluccius*	10.7 ± 4.62	[57]	
	*Diplodus vulgaris*	0.21 ± 0.03	[60]	
		0.22 ± 0.14	[53]	
	*Mullus surmuletus*	0.18 ± 0.05	[60]	
	*Lithognathus mormyrus*	0.17 ± 0.05	[60]	
		0.17 ± 0.05	[63]	
	*Dicentrarchus labrax*	8.09 ± 1.13	[61]	
	*Engraulis encrasicolus*	0.88 ± 0.01	[64]	
		1.54 ± 0.23/5.28 ± 0.85	[29]	
	*Sphyraena sphyraena*	0.80 ± 0.66/5.82 ± 0.58	[29]	
	*Sardinella aurita*	1.26 ± 0.22/3.16 ± 0.53	[29]	
	*Mugil cephalus*	0.94 ± 0.08/3.44 ± 1.40	[29]	
	*Mullus barbatus*	0.13 ± 0.04	[60]	
		0.96 ± 0.22/4.41 ± 0.93	[29]	
	*Pagellus erythrinus*	0.22 ± 0.14	[60]	
		0.21 ± 0.03	[63]	
		0.97 ± 0.15/3.03 ± 1.54	[29]	
	*Sparus aurata*	3.39 ± 0.20	[61]	
		1.31 ± 2.30	[62]	
	*Belone belone*	0.346 ± 0.040/0.454 ± 0.052	[2]	
	*Sphyraena sphyraena*	0.309 ± 0.036/0.263 ± 0.030	[2]	
	*Lophius piscatorius*	0.158 ± 0.018/0.181 ± 0.021	[2]	
Fe	*Serranus cabrilla*	80.2 ± 22.6	[57]	max. 58.685 min. 20.829
	*Diplodus sargus*	71.3 ± 20.2	[57]	
	*Sardina pilchardus*	97.2 ± 26.2	[57]	
	*Spicara maena*	37.1 ± 10.6	[57]	
	*Oblada melanura*	42.8 ± 18.9	[57]	
	*Merluccius merluccius*	115 ± 30.2	[57]	
	*Engraulis encrasicolus*	13.6 ± 0.01	[64]	
	*Sparus aurata*	3.43 ± 0.75	[62]	
	*Belone belone*	2.354 ± 0.248/5.044 ± 0.531	[2]	
	*Sphyraena sphyraena*	1.13 ± 0.090/1.936 ± 0.204	[2]	
	*Lophius piscatorius*	1.025 ± 0.250/1.244 ± 0.131	[2]	
Mn	*Serranus cabrilla*	1.44 ± 0.53	[57]	max. 1.976 min. 0.196
	*Diplodus sargus*	2.80 ± 0.48	[57]	
	*S. pilchardus*	3.62 ± 1.19	[57]	
	*Spicara maena*	0.81 ± 0.22	[57]	
	*Oblada melanura*	0.17 ± 0.07	[57]	
	*Merluccius merluccius*	1.46 ± 0.49	[57]	
	*Pomatomus saltatrix*	2.22 ± 0.15	[61]	
	*Dicentrarchus labrax*	6.79 ± 0.13	[61]	
	*Mugil cephalus*	4.16 ± 0.29	[61]	
	*Engraulis encrasicolus*	0.57 ± 0.02	[59]	
	*Sparus aurata*	4.71 ± 0.84	[61]	
		1.80 ± 1.12	[62]	
	*Lophius piscatorius*	0.182 ± 0.024/0.106 ± 0.014	[2]	
Ni	*Serranus cabrilla*	1.12 ± 0.16	[57]	max. 2.494 min. 0.172
	*Diplodus sargus*	2.90 ± 1.07	[57]	
	*S. pilchardus*	0.68 ± 0.33	[57]	
	*Spicara maena*	0.70 ± 0.17	[57]	
	*Oblada melanura*	0.27 ± 0.07	[57]	
	*Merluccius merluccius*	0.60 ± 0.19	[57]	
	*Pomatomus saltatrix*	1.85 ± 0.03	[61]	
	*Dicentrarchus labrax*	11.025 ± 0.56	[61]	
	*Mugil cephalus*	1.66 ± 0.03	[61]	
	*Sparus aurata*	4.036 ± 0.68	[61]	
	*Engraulis encrasicolus*	0.14 ± 0.22	[64]	
	*Belone belone*	0.126 ± 0.019/0.138 ± 0.021	[2]	
	*Lophius piscatorius*	0.147 ± 0.022	[2]	
Pb	*Serranus cabrilla*	0.85 ± 0.39	[57]	max. 0.429 min. 0.323
	*Diplodus sargus*	1.09 ± 0.50	[57]	
	*Sardina pilchardus*	0.90 ± 0.42	[57]	
	*Spicara maena*	0.28 ± 0.06	[57]	
	*Oblada melanura*	0.29 ± 0.04	[57]	
	*Merluccius merluccius*	1.15 ± 0.36	[57]	
		0.025	[58]	
	*Mugil soiuy*	0.078	[58]	
	*Alosa fallax*	0.046	[58]	
	*Merlangius euxmus*	0.059	[58]	
	*Scophthalmus maximus*	0.139	[58]	
	*Pomatomus saltatrix*	0.049	[58]	
	*Thunnus alalunga*	0.020–0.557	[39]	
	*Pomatomus saltatrix*	0.34 ± 0.06	[61]	
	*Dicentrarchus labrax*	0.04	[58]	
		0.11 ± 0.008	[61]	
	*Engraulis encrasicolus*	0.70 ± 0.37	[29]	
	*Sardinella aurita*	0.41 ± 0.28/0.46 ± 0.23	[29]	
	*Sphyraena sphyraena*	0.21 ± 0.10/0.98 ± 0.54	[29]	
	*Mugil cephalus*	2.34 ± 0.04	[61]	
		0.28 ± 0.10/1.30 ± 0.49	[29]	
	*Mullus barbatus*	0.0099	[58]	
		0.10 ± 0.02	[60]	
		0.21 ± 0.10/0.78 ± 0.19	[29]	
	*Mullus surmuletus*	0.10 ± 0.02	[60]	
	*Lithognathus mormyrus*	0.10 ± 0.02	[60]	
		0.11 ± 0.03	[63]	
	*Pagellus erythrinus*	0.10 ± 0.02	[60]	
		0.10 ± 0.02	[63]	
		0.20 ± 0.15/0.92 ± 0.15	[29]	
	*Diplodus vulgaris*	0.10 ± 0.02	[60]	
		0.12 ± 0.05	[63]	
	*Sparus aurata*	0.077	[63]	
		2.38 ± 0.14	[61]	
Zn	*Serranus cabrilla*	7.76 ± 1.08	[57]	max. 5.741 min. 2.582
	*Diplodus sargus*	21.7 ± 7.07	[57]	
	*Sardina pilchardus*	10.9 ± 3.80	[57]	
	*Spicara maena*	4.54 ± 0.39	[57]	
	*Oblada melanura*	3.74 ± 0.37	[57]	
	*Merluccius merluccius*	9.80 ± 1.57	[57]	
	*Lithognathus mormyrus*	5.01 ± 0.15	[63]	
	*Diplodus vulgaris*	4.95 ± 0.16	[63]	
	*Pagellus erythrinus*	5.04 ± 0.27	[63]	
		18.94 ± 2.55/32.64 ± 3.99	[29]	
	*Pomatomus saltatrix*	64.94 ± 9.09	[61]	
	*Dicentrarchus labrax*	59.25 ± 7.70	[61]	
	*Engraulis encrasicolus*	20.03 ± 0.11	[64]	
		39.44 ± 2.18/41.60 ± 8.41	[29]	
	*Sardinella aurita*	26.06 ± 6.05/32.30 ± 1.42	[29]	
	*Sphyraena sphyraena*	13.09 ± 1.19/21.15 ± 3.01	[29]	
	*Mugil cephalus*	50.14 ± 7.01	[61]	
		25.63 ± 2.43/39.69 ± 6.01	[29]	
	*Mullus barbatus*	26.63 ± 4.89/41.38 ± 2.67	[29]	
	*Sparus aurata*	67.09 ± 9.39	[61]	
		1.01 ± 0.24	[62]	
	*Belone belone*	7.071 ± 0.768/9.731 ± 1.057	[2]	
	*Sphyraena sphyraena*	3.297 ± 0.358/3.702 ± 0.402	[2]	
	*Lophius piscatorius*	3.999 ± 0.434/7.958 ± 0.864	[2]	
Hg	*Mullus surmuletus*	0.09 ± 0.03	[60]	max. 1.482 min. 0.074
	*Lithognathus mormyrus*	0.10 ± 0.03	[60]	
		0.10 ± 0.03	[63]	
	*Pagellus erythrinus*	0.09 ± 0.03	[60]	
		0.09 ± 0.03	[63]	
	*Diplodus vulgaris*	0.09 ± 0.02	[60]	
		0.09 ± 0.03	[63]	
	*Mugil soiuy*	0.014	[58]	
	*Alosa fallax*	0.028	[58]	
	*Merluccius merluccius*	0.034	[58]	
	*Merlangius euxmus*	0.063	[58]	
	*Scophthalmus maximus*	0.045	[58]	
	*Pomatomus saltatrix*	0.025	[58]	
	*Thunnus alalunga*	0.141–0.938	[39]	
	*Belone belone*	0.025 ± 0.005/0.024 ± 0.005	[2]	
	*Sphyraena sphyraena*	0.069 ± 0.014/0.088 ± 0.018	[2]	
	*Lophius piscatorius*	1.109 ± 0.225/0.211 ± 0.043	[2]	

**Table 5:** Estimated weekly intake (EWI—mg/week/70 kg body weight) is compared with provisional tolerable weekly intake (PTWI—mg/week/kg body weight).

		**Cd**	**Cr**	**Cu**	**Fe**	**Hg**	**Mn**	**Ni**	**Pb**	**Se**	**Zn**
	**PTWI**	0.007	0.0233	3.5	5.6	0.025	2.5	0.035	0.025	0.066	7
					**EWI**						
♀	Autumn	0.0139	0.0705	0.1784	9.6616	0.0434	0.2946	0.0702	0.1069	0.1681	1.3922
♂		0.0126	0.1569	0.1629	11.4036	0.1072	0.3495	0.2135	0.1258	0.2059	1.1651
♀	Winter	0.0109	0.0606	0.0811	18.9600	0.0245	0.1850	0.0569	0.1228	0.1000	0.8546
♂		0.0129	0.0788	0.0947	19.4247	0.1258	0.2284	0.8288	0.1311	0.1314	0.9288
♀	Spring	0.0113	0.1513	0.3234	19.4198	0.0520	0.6541	0.4846	0.1420	0.1509	1.1429
♂		0.0146	0.0477	0.0976	16.3646	0.0351	0.1910	0.0629	0.1337	0.1645	1.9003
♀	Summer	0.0089	0.0321	0.0784	6.8944	0.4905	0.0649	0.2287	0.1155	0.2089	1.1671
♂		0.0129	0.0705	0.2003	15.8211	0.1440	0.3313	0.3055	0.1245	0.3151	0.9781

♀: female; ♂: male.

**Table 6:** Target hazard quotient (THQ) and total THQ (∑THQ) values for heavy metals are presented for the muscle tissue of male and female scorpionfish.

		**Cd**	**Cr**	**Cu**	**Fe**	**Hg**	**Mn**	**Ni**	**Pb**	**Se**	**Zn**	
	THQ											∑THQ
♀	Autmun	1.986 ×10^−3^	3.3572 × 10^−3^	6.3717 ×10^−4^	1.9717 ×10^−3^	6.1944 ×10^−2^	3.0060 ×10^−4^	5.0122 ×10^−4^	4.3637 ×10^−3^	4.8042 ×10^−3^	6.6294 ×10^−4^	0.0805
♂		1.7968 ×10^−3^	7.4711 × 10^−3^	5.8161 ×10^−4^	2.3272 ×10^−3^	1.5320 ×10^−1^	3.5666 ×10^−4^	1.5249 ×10^−3^	5.1338 ×10^−3^	5.8823 ×10^−3^	5.5481 ×10^−4^	0.1788
♀	Winter	1.5604 ×10^−3^	2.8844 × 10^−3^	2.8962 ×10^−4^	3.8693 ×10^−3^	3.4991 ×10^−2^	1.8880 ×10^−4^	4.0665 ×10^−4^	5.0122 ×10^−3^	2.8560 ×10^−3^	4.0697 ×10^−4^	0.0525
♂		1.8441 ×10^−3^	3.7513 × 10^−3^	3.3809 ×10^−4^	3.9642 ×10^−3^	1.7968 ×10^−1^	2.3305 ×10^−4^	5.9201 ×10^−3^	5.3500 ×10^−3^	3.7544 ×10^−3^	4.4227 ×10^−4^	0.2053
♀	Spring	1.6077 ×10^−3^	7.2031 × 10^−3^	1.1549 ×10^−3^	3.9632 ×10^−3^	7.42381 ×10^−2^	6.6740 ×10^−4^	3.4613 ×10^−3^	5.7958 ×10^−3^	4.3124 ×10^−3^	5.4425 ×10^−4^	0.1029
♂		2.0805 ×10^−3^	2.2697 × 10^−3^	3.4873 ×10^−4^	3.3397 ×10^−3^	5.0122 ×10^−2^	1.9488 ×10^−4^	4.4921 ×10^−4^	5.4581 ×10^−3^	4.7002 ×10^−3^	9.0489 ×10^−4^	0.0699
♀	Summer	1.2767 ×10^−3^	1.5289 × 10^−3^	2.8016 ×10^−4^	1.4070 ×10^−3^	7.0077 ×10^−1^	6.62 E−5	1.6337 ×10^−3^	4.7150 ×10^−3^	5.9674 ×10^−3^	5.5576 ×10^−4^	0.7182
♂		1.8441 ×10^−3^	3.3572 × 10^−3^	7.1519 ×10^−4^	3.2288 ×10^−3^	2.0569 ×10^−1^	3.3809 ×10^−4^	2.1822 ×10^−3^	5.0798 ×10^−3^	9.0032 ×10^−3^	4.6576 ×10^−4^	0.2319

♀: female; ♂: male.

**Table 7:** The calculated CR (carcinogenic risk) levels in male and female scorpionfish.

Gender	Seasons	Cd	Cr	Ni	Pb
♀	Autumn	7.5 × 10^−4^	5.0 × 10^−3^	9.1 × 10^−3^	1.3 × 10^−4^
♂	Autumn	6.8 × 10^−4^	1.12 × 10^−2^	2.78 × 10^−2^	1.5 × 10^−4^
♀	Winter	5.9 × 10^−4^	4.3 × 10^−3^	7.4 × 10^−3^	1.5 × 10^−4^
♂	Winter	7.0 × 10^−4^	5.6 × 10^−3^	1.08 × 10^−1^	1.6 × 10^−4^
♀	Spring	6.1 × 10^−4^	1.08 × 10^−2^	6.30 × 10^−2^	1.7 × 10^−4^
♂	Spring	7.9 × 10^−4^	3.4 × 10^−3^	8.2 × 10^−3^	1.6 × 10^−4^
♀	Summer	4.9 × 10^−4^	2.3 × 10^−3^	2.97 × 10^−2^	1.4 × 10^−4^
♂	Summer	7.0 × 10^−4^	5.0 × 10^−3^	3.97 × 10^−2^	1.5 × 10^−4^

♀: female; ♂: male.

**Table 8:** Distribution of MPI and HBVSe values across seasons and genders.

Gender	Seasons	MPI	HBV_Se_
♀	Autumn	0.547	0.46
♂	Autumn	0.750	0.45
♀	Winter	0.421	0.28
♂	Winter	0.734	0.02
♀	Spring	0.878	0.40
♂	Spring	0.512	0.47
♀	Summer	0.538	−1.63
♂	Summer	0.796	0.76

♀: female; ♂: male.

## Data Availability

The original contributions presented in this study are included in the article. Further inquiries can be directed to the corresponding author.
